# Eggs for the Sick

**Published:** 1871-07

**Authors:** 


### EGGS FOR THE SICK.

Fresh eggs are a great comfort to some invalids. As soon as possible after an egg is laid and cooled, dip it in a vessel of glycerine, or gum arabic dissolved with water to the consistence of milk as it comes from the cow; pack them away in charcoal dust or fine, dry sand, in a cool, dry cellar, and they will remain almost as good as fresh for many weeks.

